# Three new caespitose species of *Senecio* (Asteraceae, Senecioneae) from South Peru

**DOI:** 10.3897/phytokeys.39.7668

**Published:** 2014-06-19

**Authors:** Daniel B. Montesinos Tubée

**Affiliations:** 1 Nature Conservation & Plant Ecology Group, Wageningen University, Netherlands. Droevendaalsesteeg 3a, 6708PB Wageningen, The Netherlands; 2 Naturalis Biodiversity Centre, Botany Section, N; 3 ational Herbarium of The Netherlands, Herbarium Vadense. Darwinweg 2, 2333 CR Leiden, The Netherlands; 4 Instituto Científico Michael Owen Dillon, Av. Jorge Chávez 610, Cercado, Arequipa, Perú

**Keywords:** Compositae, new species, *Senecio* subser. *Caespitosi*, South America, taxonomy

## Abstract

Three new species of the genus *Senecio* (Asteraceae, Senecioneae) belonging to *Senecio* ser. *Suffruticosi* subser. *Caespitosi* were discovered in the tributaries of the upper Tambo River, Moquegua Department, South Peru. Descriptions, diagnoses and discussions about their distribution, a table with the morphological similarities with other species of *Senecio*, a distribution map, conservation status assessments, and a key to the caespitose Peruvian species of *Senecio* subser. *Caespitosi* are provided. The new species are *Senecio moqueguensis* Montesinos, **sp. nov.** (Critically Endangered) which most closely resembles *Senecio pucapampaensis* Beltrán, *Senecio sykorae* Montesinos, **sp. nov.** (Critically Endangered) which most closely resembles *Senecio gamolepis* Cabrera, and *Senecio tassaensis* Montesinos, **sp. nov.** (Critically Endangered) which most closely resembles *Senecio moqueguensis* Montesinos.

## Abstract (es)

Tres nuevas especies del género *Senecio* (Asteraceae, Senecioneae) pertenecientes a *Senecio* ser. *Suffuticosi* subser. *Caespitosi* fueron descubiertas en las alturas de la cuenca del río Alto Tambo, Departamento Moquegua, Sur de Perú. Las especies se describen y tipifican, una diagnosis y discusión acerca de su distribución, una tabla con las similitudes morfológicas con otras especies de *Senecio*, un mapa de distribución, el estatus de conservación y una clave para las especies peruanas cespitosas de *Senecio* subser. *Caespitosi* son presentadas. Las nuevas especies son *Senecio moqueguensis* Montesinos, **sp. nov.** (En Peligro Crítico) el cual se asemeja más a *Senecio pucapampaensis* Beltrán, *Senecio sykorae* Montesinos, **sp. nov.** (En Peligro Crítico) el cual se asemeja más *Senecio gamolepis* Cabrera, y *Senecio tassaensis* Montesinos, **sp. nov.** (En Peligro Crítico) el cual se asemeja más a *Senecio moqueguensis* Montesinos.

## Introduction

*Senecio* contains about 175 species in Peru (Brako and Zaruchi 1993, Vision and Dillon 1996) including several recently described new species (Beltrán 2009). The genus has 94 species endemic to Peru which have been evaluated and classified according to IUCN criteria (Beltrán et al. 2007). In the Department of Moquegua, 30 species have been recorded (Arakaki and Cano 2003, Montesinos 2012). The species of *Senecio* described here were discovered in the tributaries of the upper Tambo River in southern Peru, an area of extraordinary species richness and a high level of endemism (Montesinos 2011, 2012).

*Senecio* ser. *Suffruticosi* Cabrera accounts for 143 species occurring on the American continent, especially in the Andes and Patagonia (Cabrera 1949, Cabrera 1985, Cabrera et al. 1999). Cabrera et al. (1999) divided *Senecio* ser. *Suffruticosi* into five subseries and described it as embracing suffruticose or perennial herbs, glabrous or glandulose, with entire leaves which are dentate or, more rarely, incised, involucres discoid, and capitula isomorphic. Among those subseries, *Senecio* subser. *Caespitosi* Cabrera contains 50 species (Cabrera et al. 1999), of which thirteen occur in Peru at altitudes between 3500 m and 5000 m (Brako and Zaruchi 1993, Beltrán et al. 2007): *Senecio adenophyllus* Meyen & Walp., *Senecio algens* Wedd., *Senecio scorzonerifolius* Meyen & Walp. and *Senecio trifurcifolius* Hieron. also distributed in northwestern Argentina, Bolivia and north of Chile, *Senecio danai* A. Gray and *Senecio pucapampaensis* Beltrán occurring only in central Peru (Beltrán et al. 2007), *Senecio evacoides* Sch. Bip., *Senecio expansus* Wedd. and *Senecio humillimus* Sch. Bip. also distributed in northwestern Argentina and Bolivia, *Senecio gamolepis* Cabrera, endemic to central and southern Peru, *Senecio rufescens* DC. distributed from Colombia to northwestern Argentina, *Senecio repens* Stokes distributed from south Ecuador through Peru and northwestern Bolivia, and *Senecio vegetus* (Wedd.) Cabrera, also distributed in Bolivia. In *Senecio* subser. *Caespitosi* plants are characterized as suffruticose (or herbaceous), glabrous or glandulose; leaves entire, dentate or, more rarely, incised; capitula discoid, medium or small; and flowers isomorphic (Cabrera et al. 1999).

Notwithstanding the progress in taxonomical and molecular studies (Nordenstam 1977, Cabrera 1949, 1985, Cabrera et al. 1999, Pelser et al. 2007, Nordenstam et al. 2009), there are more species of the tribe *Senecioneae* occurring in the Andes which remain poorly understood and are awaiting discovery. Intergeneric relationships within Senecioneae are still largely unknown (Pelser et al. 2007); furthermore, the lack of knowledge about generic-level evolutionary relationships in Senecioneae remains the largest taxonomic problem on the way to obtaining a monophyletic delimitation of *Senecio* (Bremer 1994, Pelser et al. 2007). Phylogenetic positions for the members of *Senecio* subser. *Caespitosi* are still largely unknown, except for *Senecio algens*, *Senecio humillimus* and *Senecio rufescens* (Pelser et al. 2007), of which *Senecio algens* belongs to the *Aetheolaena involucrata*-*Aetheolaena patens* clade and *Senecio humillimus* and *Senecio rufescens* to the *Senecio glaber*-*Senecio donianus* clade. Numerous new collections from Moquegua have been made in recent years (Montesinos 2011, 2012). A comparison with herbarium specimens, together with a review of the literature and taxonomic keys, has shown that these collections include three new species of *Senecio* subser. *Caespitosi* which are described below. These new species were separated from the other species of this subseries on the basis of a set of characters such as habit, the presence or absence of trichomes, flower color, the number of phyllaries and involucral bracts, the involucre length and the achene type (Cabrera 1955, 1985, Cabrera et al. 1999). The new species can be found at elevations above 4500 m as terrestrial plants on bare rocky soils on the summits of high mountains in the north of Moquegua department, where they co-occur with several other acaulescent *Senecioneae* from *Senecio* subser. *Caespitosi* such as *Senecio gamolepis*, *Senecio evacoides*, and *Senecio algens*.

## Methods

Based on morphological characters, an overview of the genus *Senecio* with an emphasis on *Senecio* subser. *Caespitosi* from Peru and adjacent areas (Ecuador, Bolivia, Argentina and northern Chile) has been prepared, based on Cabrera (1955, 1985) and Cabrera et al. (1999). Since 2009 I have examined more than 450 specimens of *Senecio* subser. *Caespitosi* housed in Peruvian herbaria (CUZ, HSP, HUPCH, HUSA, MOL, USM), relevant collections from institutions abroad (B, BR, F, L, LPB, MO, P, WAG), and material from my recent fieldwork. Digitised specimens were viewed via online herbarium catalogues (http://tropicos.org and http://fm1.fieldmuseum.org/vrrc/) or via JSTOR (2013). All morphological characters were studied under a NSZ-405 1X-4.5X stereo microscope and an AmScope M100C-LED 40×-1000× compound microscope. The descriptions were made using the terminology presented by Cabrera (1955, 1985), Cabrera et al. (1999), Vision and Dillon (1996), Beltrán (2009), Nordenstam et al. (2009) and Roque et al. (2009). Conservation assessments were undertaken using the IUCN criteria (IUCN 2001).

## Taxonomy

### Senecio moqueguensis

Taxon classificationPlantaeAsteralesAsteraceae

Montesinos sp. nov.

urn:lsid:ipni.org:names:77140249-1

Figs 1 , 4A , 5

#### Diagnosis.

The new species is morphologically similar to *Senecio pucapampaensis* but is clearly distinguished by the leaf lamina oblong-spathulate (vs. cuneiform), leaf surface covered by thin trichomes (vs. glabrous), corolla yellow (vs. white), calycular bracts linear-oblong, 6–9 mm long (vs. linear, 6–7 mm long), and phyllaries 9–12 (vs. 12–14).

#### Type.

PERU. Moquegua Region, General Sánchez Cerro Province, Ubinas District, NW of Tassa, terrestrial on clayey rocky soils on the plateau peaks near Lake Pacosani, elevation 4653 m, 16°06'43"S, 70°44'45"W, 3 April 2009, *Montesinos 2400* (holotype USM!, isotypes MO 2383567, HUPCH 4185, CPUN, WAG 0246107).

#### Description.

*Perennial* herb, rhizomatous, creeping, low-growing, forming mats 2–4 cm tall and up to 60 cm in diam. *Trichomes* glandular, somewhat dense and irregularly distributed, multicellular, whitish transparent, 0.1–0.3 mm long and 0.05–0.1 mm wide and composed of 4–8 subrotund cells (each 30–50 µm long), apical cell rotund. *Stems* 1–3 cm long, often densely branched and leafy in the central part, rooting. *Leaves* cauline, lamina oblong-spathulate, 8–12 mm × 1–2.5 mm, sparsely covered by thin trichomes on the margins, lower and upper surfaces except at the base; base truncated, apex subpinnatifid; young leaves dark green with yellowish margins, turning light green-greyish with age. *Synflorescences* of solitary sessile or subsessile terminal capitula. *Capitula* homogamous, discoid. *Involucres* at first broadly cylindrical, gradually turning campanulate with age, ca. 7–10 mm long × 6–8.5 mm wide). *Calycular bracts* linear-oblong (6–9 mm × 1–2.5 mm), whitish green on the surface and whitish along the margins, with scarce trichomes near the midrib and margins, apex dark brown covered with short light-brown trichomes. *Phyllaries* 9–12, connate, 5–8 mm long × 0.7–1.2 mm wide, oblong-lanceolate, covered with thin trichomes sparsely on the surface and densely along the margins, apex greenish grey and dark brown with short white multicellular trichomes. *Florets* 24–28; corolla tubular, abruptly constricted near the base, 5-lobed, each lobe 0.5 mm long, bright yellow, tube 3–5 mm long × 0.8–1 mm wide; anthers linear-lanceolate, 1.5–2.5 mm long, 0.2–0.4 mm wide, ecalcarate, terminal appendages lanceolate, obtuse, margin whitish transparent and becoming yellow towards the centre; style dark yellow, truncate, papillae covering the whole surface of the apex. *Achenes* cylindrical, covered with trichomes, 1.8–2.5 mm long and 0.4–0.8 mm wide, light brown; carpopodium symmetrical in a small ring; pappus of smooth bristles, white, silky, 5–6 mm long, with fine single setulae.

**Figure 1.**
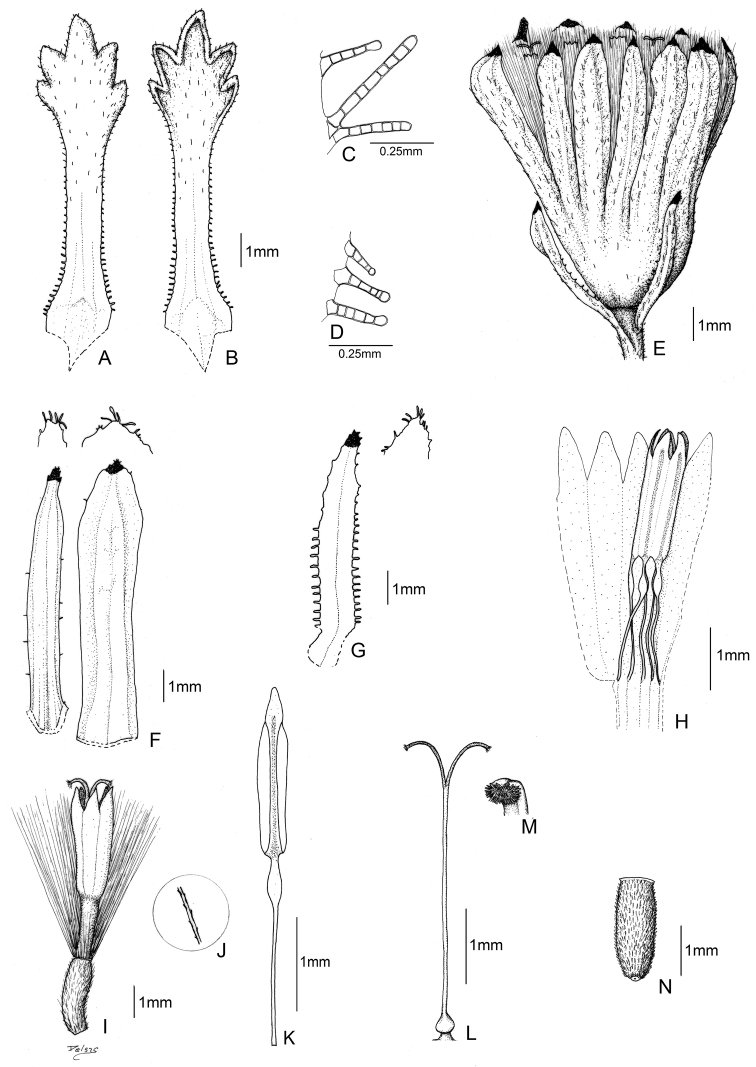
*Senecio moqueguensis* Montesinos. **A** Leaf (upper side) **B** Leaf (underside) **C** Phyllary trichomes **D** Leaf trichomes **E** Capitulum **F** Calycular bracts **G** Phyllary **H** Stamens arrangement in a floret **I** Floret **J** Pappus bristles **K** Stamens **L** Style **M** Papillose stigma **N** Achene

#### Ecology and distribution.

Terrestrial plant on clayey rocky soils on the peaks of the highland summits and grasslands in the north of Moquegua Region, at elevations of ca. 4500 to 4800 m. Co-occurring species include *Azorella compacta* Phil., *Calamagrostis vicunarum* (Wedd.) Pilg., *Pycnophyllum molle* Remy, and *Festuca* spp. Flowers and fruits between March and April.

#### Etymology.

The specific epithet refers to Moquegua, where the only three collections are known from the north of the department.

#### Additional material examined 

**(paratypes).** PERU. Moquegua Region, General Sánchez Cerro Province, Ubinas District, terrestrial on bare clayey soils in the verges of the road east Pillone town, elevation 4584 m, 16°10'02"S, 70°49'56"W, 24 March 2013, *Montesinos 4022* (USM, HUSA). Moquegua Region, General Sánchez Cerro Province, Ubinas District, NW of Tassa, terrestrial on bare clayey soils in the verges of the road to Lake Cochapata, elevation 4687 m, 16°08'56"S, 70°43'0.30"W, 9 December 2013, *Montesinos 4200* (CUZ).

#### Discussion.

A comparison of the material has shown that *Senecio moqueguensis* is most similar to *Senecio pucapampaensis* and *Senecio tassaensis* sp. nov. Together with *Senecio evacoides*, *Senecio expansus*, *Senecio repens* and *Senecio humillimus*, it forms a coherent morphological and geographical group within *Senecio* subser. *Caespitosi* which occurs from central Peru to northwest Argentina and is characterized by the presence of trichomes on stems, leaves and involucres. *Senecio moqueguensis* can be distinguished from *Senecio pucapampaensis* by the dense caespitose mat habit, leaves, calycular bracts, corolla color, involucres and achene morphology as summarised in Table 1. *Senecio moqueguensis* can be distinguished from *Senecio evacoides*, *Senecio expansus* and *Senecio repens* by the habit, density of trichomes, leaf shape and length, as well as by the calycular bracts and phyllary length and form.

**Table 1.** Comparison between *Senecio moqueguensis*, *Senecio sykorae*, *Senecio tassaensis* and their closest relatives.

	***Senecio moqueguensis***	***Senecio sykorae***	***Senecio tassaensis***	***Senecio pucapampaensis***	***Senecio gamolepis***	***Senecio algens***	***Senecio evacoides***	***Senecio humillimus***	***Senecio expansus***	***Senecio trifurcifolius***	***Senecio repens***
**Distribution**	PE (Moquegua)	PE (Moquegua)	PE (Moquegua)	PE (Junin)	PE, CH, AR, BO	PE, BO, AR	PE, BO, AR	BO, South Peru	AR, BO, PE	PE, BO, CH	BO, EC, PE
**Altitude**	4500–4800 m	4550–4800 m	4650–4700 m	4500–4600 m	4000–4800 m	4500–5000 m	4000–4800 m	3500–4500 m	3900–4800 m	4000–4500 m	3000–4600 m
**Habit**	dense caespitose mat	tuft mat	tuft	postrate, decumbent	dense caespitose mat	caespitose subshrub	suffruticose or shrubby	dense caespitose mat	ground rosette herb	suffruticose	ground rosette herb
**Plant dimensions (height, diameter)**	2–4 cm, > 60 cm	4–6 cm, > 6 cm	2–4 cm, > 4 cm	5–9 cm, > 8 cm	2–3 cm, > 1 m	4–6 cm, > 6 cm	2 cm, ca. 1 m	2 cm, ca. 70 cm	2–4 cm, 5–8 cm	5–8 cm, > 8 cm	2–4 cm, 6–9 cm
**Indumentum**	glandular, multicelular, 0.1–0.3 mm	absent	glandular, multicelular, 0.3–1.2 mm	finely puberulous, <0.1 mm	absent	absent	white lanuginose, < 0.2 mm	puberulous, 0.1–0.2 mm	densely lanuginose, < 0.2 mm	absent	puberulous, < 0.2 mm
**Leaf shape**	oblong-spathulate, subpinnatifid	obovate-spathulate	obovate-spathulate, incised or acuminate	cuneiform-subpinnatifid, incised	linear-lanceolate	spathulate, obtuse	obovate-spathulate	linear-spathulate, ovate	ovate, elliptical or circular, crenate	cuneiform-linear, dentate	elliptic-ovate, obovate
**Leaf (length, width)**	8–12 × 1–2.5 mm	9–14 × 1–2.2 mm	6–9 × 1–2.5 mm	9–15 × 3–4 mm	8–12 × 2–4 mm	10–35 × 2–5 mm	10–20 × 3–6 mm	3–10 × 0.5–1 mm	10–25 × 10–22 mm	10–20 × 1 mm	10–25 × 10–22 mm
**Leaf pubescence**	sparsely covered by thin trichomes	glabrous	densely covered by trichomes	ciliate margins	glabrous	glabrous	densely lanuginose	sparsely puberulous	densely lanuginose	glabrous	glabrous adaxially, puberulous abaxially
**Involucre (shape; length; width)**	cylindrical-campanulate; 7–10 × 6–8.5 mm	cylindrical-campanulate; 7–9 × 3–5 mm	cylindrical-campanulate; 6–8 × 5–7 mm	campanulate; 7–8 × 8–9 mm	cylindrical-campanulate; 8–11 × 4–6 mm	cylindrical-campanulate; 7.5–10 × 8–12 mm	campanulate; 7–8 × 5–6 mm	cylindrical-campanulate; 5 × 3–4 mm	campanulate; 10–25 × 10–22 mm	campanulate; 8–9 × 6 mm	campanulate; 6–7 × 8–10 mm
**Calycular bracts (shape; margin; size)**	linear-oblong; sparse trichomes; 6–9 × 1–2.5 mm	linear-oblong; scarce trichomes; 6–8 × 0.7–1 mm	ovate-oblong; dense trichomes; 4–6 × 1 mm	linear; ciliate; 6–7 × 1 mm	linear; glabrous; 7–10 × 0.8–1.2 mm	linear; glabrous; 6–9 × 0.8–1.1 mm	linear; tomentose; 6–7 × 0.8–1.2 mm	linear; glabrous; 3–4 × 0.7–1 mm	linear; tomentose; 9–22 × 2.5–5 mm	linear, glabrous; 7–8 × 0.8–1.2 mm	linear, glabrous; 5–6 × 0.8–1.2 mm
**Phyllaries (shape; size)**	oblong-lanceolate; 5–8 × 0.7–1.2 mm	linear-lanceolate; 5–6.5 × 0.6–1 mm	linear-lanceolate; 5–8 × 0.8–1.2 mm	linear; 6–7 × 1.2 mm	oblong; 6–8 × 1.8–2.3 mm	linear; 7–9 × 2–3 mm	linear; 5–7 × 0.8–1.1 mm	oblong-lanceolate; 6–8 × 1–1.2 mm	linear; 10–15 × 2–4 mm	lanceolate, attenuate; 6–8 × 1–1.5 mm	linear; 13–20 mm × 1–2 mm
**Phyllaries (number)**	9–12	12–14	12–16	12–14	7–9	10–15	13–20	8	20–25	8	13–20
**Phyllaries (margins)**	densely covered with trichomes	glabrous	densely covered with trichomes	scarious, ciliate	glabrous	glabrous	pubescent	glabrous	glabrous or pubescent	glabrous	glabrous
**Corolla (color)**	bright yellow	white	purple-pink to pale white	white	yellow	yellow	yellow	dark yellow with purple tube	yellow	yellow	yellow
**Achene (shape, texture)**	cylindrical, with trichomes	cylindrical, with trichomes	ovate, striate, with trichomes	cylindrical, glabrous	cylindrical, glabrous	cylindrical, glabrous	cylindrical, glabrous	cylindrical, sericeous	cylindrical, glabrous	cylindrical-ovate, densely sericeous	cylindrical, glabrous
**Pappus (length)**	5–6 mm	4–6 mm	3.5–5 mm	5–6 mm	6–9 mm	6–8 mm	4–6 mm	5–7 mm	10–20 mm	6–7 mm	5–6 mm

#### Conservation status.

Following the criteria and categories of IUCN (2001), a preliminary status of Critically Endangered (CR) is assigned. The new species deserves protection because its total area of occupancy is less than 100 km² (ca. 50 km²) (B1); only three populations are known (B1b); habitat inferred to be continuing to decline (B1b(i-iii)); population estimated to number fewer than 300 individuals (D). The suitable habitats for *Senecio moqueguensis* on the mountain summits near the set of lakes in the Ubinas district are regarded as endangered because overgrazing of grasslands, changes in annual rainfall, volcanic activity, and exploitation of natural resources may all potentially reduce their extent.

### Senecio sykorae

Taxon classificationPlantaeAsteralesAsteraceae

Montesinos sp. nov.

urn:lsid:ipni.org:names:77140250-1

Figs 2 , 4B , 5

#### Diagnosis.

Morphologically similar to *Senecio gamolepis* but clearly distinguished by the tuft mat habit (vs. cushion mats), the leaf shape being obovate-spathulate (vs. linear-lanceolate), corolla white (vs. yellow), phyllaries 12–14 (vs. 7–9), disc length 7–9 mm (vs. 8–12 mm), and achene length 1.5–2 mm (vs. 1–1.3 mm).

#### Type.

PERU: Moquegua Region, General Sánchez Cerro Province, Yunga District, E of Yunga, terrestrial on bare clayey soils on the peaks of Perusa mountain, elevation 4802 m, 16°11'08"S, 70°38'14"W, 13 April 2012, *Montesinos & Calisaya 3805* (holotype USM!, isotype HUSA!).

#### Description.

*Perennial* herb, decumbent, low-growing and forming small tuft mats 4–6 cm high and up to 6 cm in diam. *Trichomes* absent. *Stems* 3–5 cm long, densely leafy, woody and branched at the base. *Leaves* cauline, alternate, lamina obovate-spathulate, 9–14 mm long × 1–2.2 mm wide, glabrous on surface and margins except at the base (scarcely covered by thin, short trichomes), base truncate to auriculate, apex obtuse, entire, margin involute; young leaves pale green with yellowish margins turning dark green with age. *Synflorescences* of solitary, terminal capitula. *Capitula* homogamous, discoid and pedicled (5–10 mm long). *Involucres* at first narrowly cylindrical becoming cylindrical-campanulate with age (7–9 mm long × 3–5 mm wide). *Calycular bracts* linear-oblong (6–8 mm × 0.7–1 mm), dark green on the surface and light green along the margins, with dark brown-black apex covered with inconspicuous trichomes or glabrous. *Phyllaries* 12–14, connate, 5–6.5 mm long × 0.6–1 mm wide, linear-lanceolate, margins glabrous, apex dark brown with short trichomes. *Florets* 13–16; corolla tubular, abruptly constricted near the base, 5-lobed, each lobe 0.2–0.3 mm long, white, tube 2.5–4 mm long × 0.5–0.8 mm wide; anthers linear-lanceolate, 1.5–2 mm long × 0.2–0.3 mm wide, truncate, terminal appendages lanceolate, obtuse; margin whitish transparent and becoming darker towards the centre; style dark purple, truncate, apically covered by papillae equally distributed. *Achenes* cylindrical, pale green, finely covered with trichomes, 1.5–2 mm long × 0.6–0.9 mm wide; carpopodium symmetrical in a shallow ring; pappus of smooth fine bristles, white, 4–6 mm long, with fine alternate single setulae.

**Figure 2.**
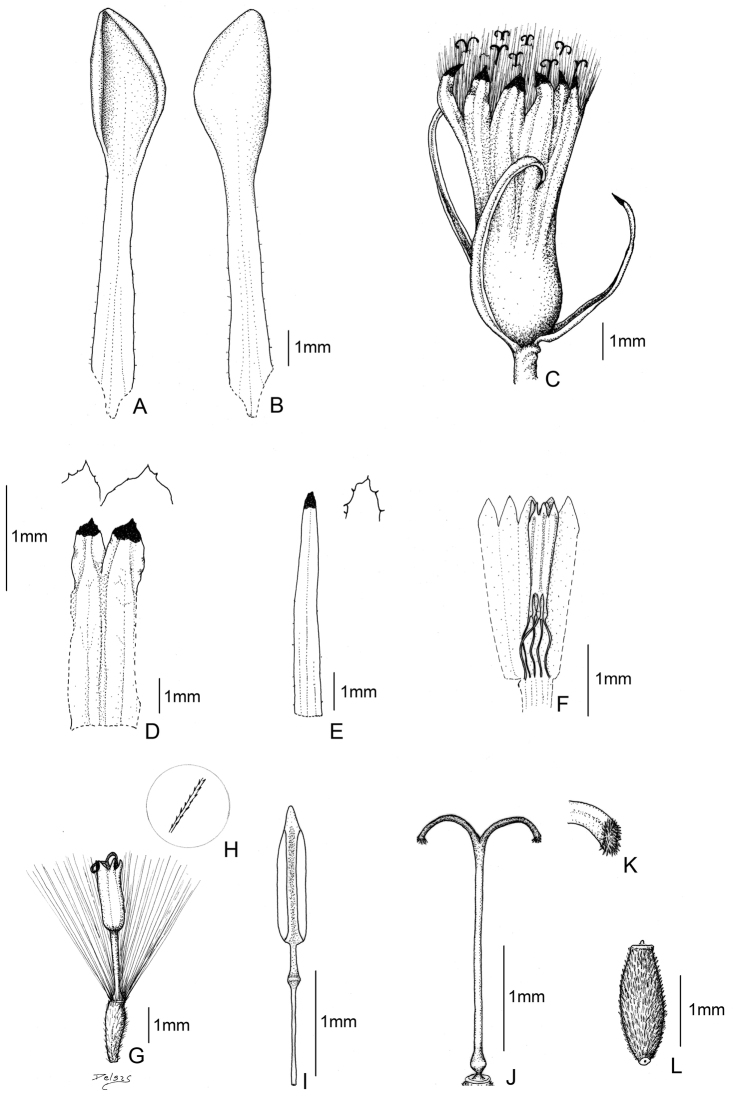
*Senecio sykorae* Montesinos. **A** Leaf (upper side) **B** Leaf (underside) **C** Capitulum **D** Calycular bracts **E** Phyllary **F** Stamens arrangement in a floret **G** Floret **H** Pappus bristles **I** Stamens **J** Style **K** Papillose stigma **L** Achene

#### Ecology and distribution.

Terrestrial plant on bare clayey soils on the summits of mountain peaks and grassland plateaus in the north of the Moquegua Region at elevations of 4550–4800 m. Co-occurring with *Belloa pickeringii* (A. Gray) Sagást. & M.O. Dillon, *Nototriche obcuneata* (Baker f.) A.W. Hill, *Pycnophyllum molle* Remy, *Senecio candollei* Wedd. and *Xenophyllum ciliolatum* (A. Gray) V.A. Funk. Flowers and fruits between March and April.

#### Etymology.

This *Senecio* is named after Karlè Sýkora, a well-known Dutch vegetation scientist who was my mentor in phytosociology.

#### Additional material examined

**(paratypes).** PERU. Moquegua Region, General Sánchez Cerro Province, Ubinas District, S of Pillone, terrestrial on bare clayey soils in the verges of the road to Pillone town, elevation 4584 m, 16°10'02"S, 70°49'56"W, 24 March 2013, *Montesinos 4023* (USM).

#### Discussion.

*Senecio sykorae* appears to be closely related to *Senecio gamolepis* which grows at higher elevations but approaches the known range of *Senecio sykorae* within a few hundred metres. While *Senecio gamolepis* is generally distinctive in the genus for its large size, attaining widths of up to 1 meter in diameter, and for its larger, capitulate form, *Senecio sykorae* is a smaller plant, of about 4–6 cm wide and has shorter corolla, less than 9 mm long. *Senecio sykorae* is also distinctive in that it has 12–14 phyllaries per capitulum instead of 7–9 phyllaries in *Senecio gamolepis*. Likely the leaves of *Senecio sykorae* are distinctive in that they are obovate-spathulate vs. linear-lanceolate. Also, the achenes in *Senecio sykorae* are larger (1.5–2 mm long) vs. 1–1.3 mm long in *Senecio gamolepis*. *Senecio sykorae* also differs from *Senecio algens* by the leaf and capitula length (shorter in *Senecio sykorae*), and from both species by the corolla colour (white vs. yellow). Less similarity is found in *Senecio algens*, *Senecio humillimus*, *Senecio trifurcifolius*, *Senecio pucapampaensis* and *Senecio evacoides*, and from which *Senecio sykorae* can be distinguished on the basis of its habit, trichomes, leaf shape and length, calycular bracts and phyllary length and shape as summarized in Table 1.

#### Conservation status.

Following the criteria and categories of IUCN (2001), a preliminary status of Critically Endangered (CR) is assigned. The new species deserves protection because its total area of occupancy is less than 10 km² (ca. 5 km²) (B2); only one population known (B2b); habitat inferred to be continuing to decline (B2b(i-iii)); population estimated to number fewer than 150 individuals (D). The suitable habitats for *Senecio sykorae* on the mountain summits of the north of Moquegua are indicated as endangered because of overgrazing of grasslands, changes in annual rainfall, volcanic activity, and exploitation of natural resources, all potentially reducing their extent.

### Senecio tassaensis

Taxon classificationPlantaeAsteralesAsteraceae

Montesinos sp. nov.

urn:lsid:ipni.org:names:77140251-1

Figs 3 , 4C , 5

#### Diagnosis.

Similar to *Senecio moqueguensis* but clearly distinguished by the leaf lamina obovate-spathulate (vs. oblong-spathulate), leaf length 6–9 mm (vs. 8–12 mm), leaf surface densely covered by trichomes (vs. sparsely covered), trichomes 0.3–1.2 mm long (vs. 0.1–0.3 mm long), corolla white (vs. yellow), calycular bracts 4–6 mm long (vs. 6–9 mm), phyllaries 12–16 (vs. 9–12), involucre length 6–8 mm (vs. 7–10 mm), and achene length 1–1.2 mm (vs. 1.8–2.5 mm).

#### Type.

PERU. Moquegua Region, General Sánchez Cerro Province, Ubinas District, NW of Tassa, terrestrial on clayey rocky soils on the summits of Pirhuani peak, elevation 4657 m, 16°09'58"S, 70°43'49"W, 07 April 2011, *Montesinos 3103* (holotype HUSA!, isotypes MOL, USM).

#### Description.

*Perennial* herb, tufted, up to 2–4 cm high and up to 4 cm in diam. *Trichomes* glandular, densely covering the plant, multicellular, whitish transparent, 0.3–1.2 mm long × 0.1–0.2 mm wide, composed of 6–10 ovate or elongate cells (each 60–80 µm long), apical cell rotund. *Stems* thick, < 1 cm long, often densely branched and leafy in the central portion. *Leaves* arranged in irregular rosettes, lamina obovate-spathulate, 6–9 mm × 1–2.5 mm, densely covered by thin trichomes on the margins; base truncated and apex pinnatifid; lower and upper surface of the leaves gradually becoming shorter towards the tip; margin incised with 5–7 obtuse lobes or rarely acuminate; mature leaves with involute margins; young leaves green yellow turning greenish grey with age. *Synflorescences* of solitary sessile or subsessile terminal capitula. *Capitula* homogamous, discoid. *Involucres* at first cylindrical, turning campanulate with age (ca. 6–8 mm long × 5–7 mm wide). *Calycular bracts* ovate-oblong (4–6 mm × 1 mm), greyish green on the surface and covered with trichomes on the margins, dark brown apex covered apically with short brown multicellular trichomes. *Phyllaries* 12–16, connate, 5–8 mm long × 0.8–1.2 mm wide, linear-lanceolate, covered with thin trichomes scarcely on the surface and densely along the margins, apex dark brown and covered with short multicellular trichomes. *Florets* 18–21, corolla tubular, abruptly constricted near the base, 5-lobed, each lobe 0.2–0.4 mm long, purple pink gradually becoming pale white towards the tip, tube 2–2.5 mm long × 1 mm wide; anthers linear-lanceolate, 1.5–2 × 0.3–0.4 mm, terminal appendages lanceolate, acute to somewhat protuberant, bases ecalcarate; anthers margin white becoming dark yellow towards the centre; style dark purple, truncate, papillae covering the whole surface of the apex. *Achenes* ovate, striate, covered with trichomes, 1–1.2 mm long and 0.6–0.8 mm wide, pale yellow; carpopodium symmetrical in a shallow ring; pappus of smooth bristles, white, silky, 3.5–5 mm long, with fine single setulae.

**Figure 3.**
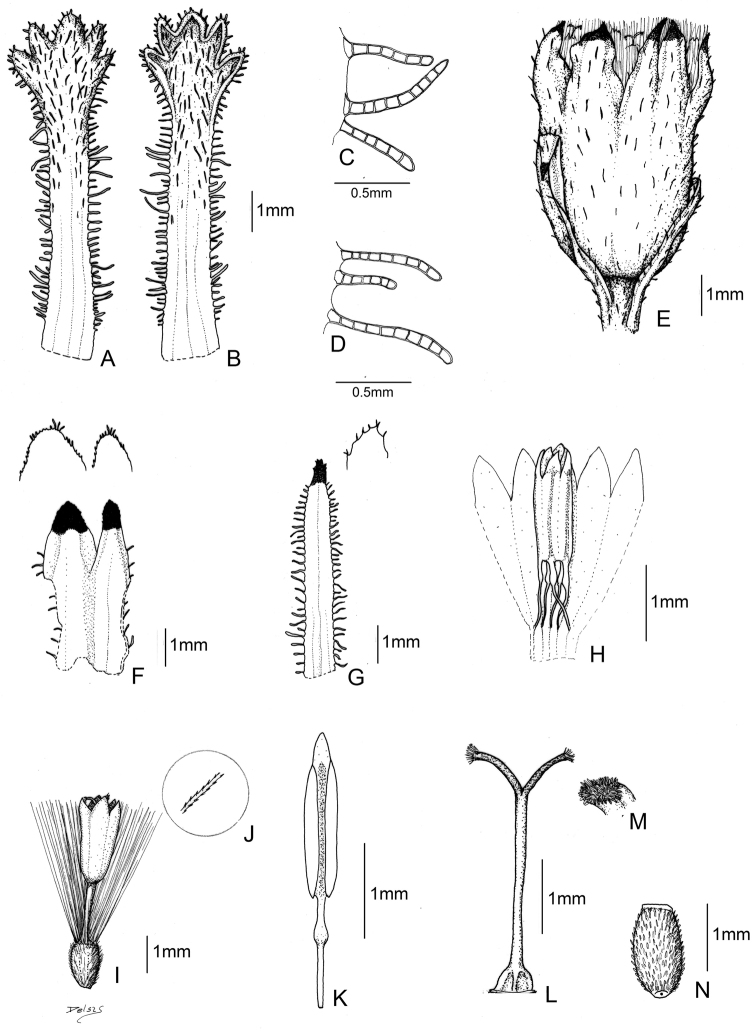
*Senecio tassaensis* Montesinos. **A** Leaf (upper side) **B** Leaf (underside) **C** Phyllaries trichomes **D** Leaf trichomes **E** Capitulum **F** Calycular bracts **G** Phyllary **H** Stamens arrangement in a floret **I** Floret **J** Pappus bristles **K** Stamens **L** Style **M** Papillose stigma **N** Achene.

**Figure 4.**
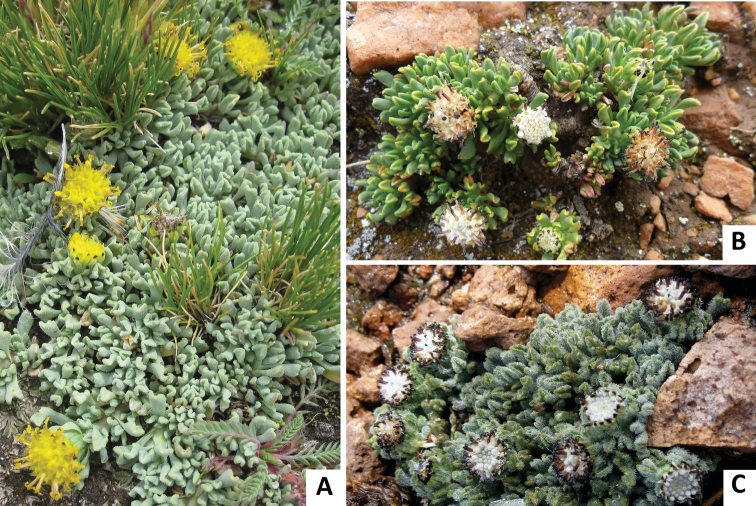
Habit photographs of: **A** *Senecio moqueguensis* **B** *Senecio sykorae* **C** *Senecio tassaensis*.

**Figure 5.**
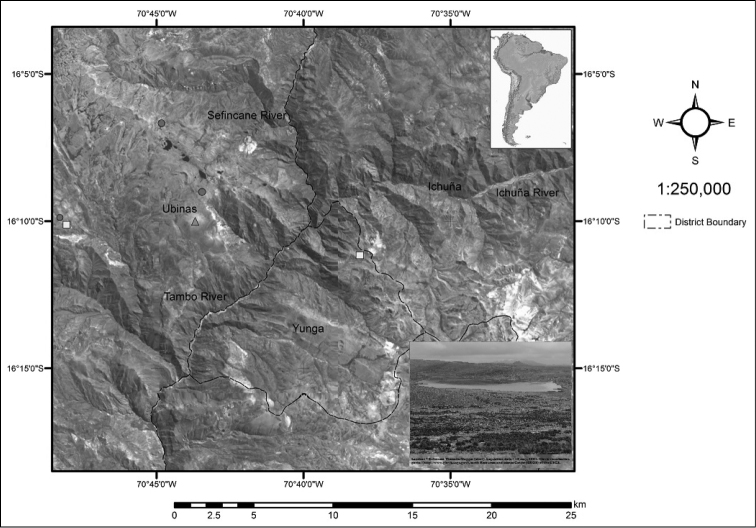
Distribution map showing collection and recorded sites for *Senecio moqueguensis* (red circles), *Senecio sykorae* (yellow squares) and *Senecio tassaensis* (green triangles). Inset: Photograph of the highland plains of Tassa, Moquegua, where populations of *Senecio moqueguensis* occur.

#### Ecology and distribution.

Terrestrial plant on clayey rocky soils on the peaks of the highland summits of the Pirhuani peak, near Tassa town, Moquegua Region, at elevations of 4650–4700 m. It occurs with *Azorella*, *Calamagrostis*, *Pycnophyllum*, *Mniodes*, *Senecio*, and *Xenophyllum*. Flowers and fruits between March and April.

#### Etymology.

This *Senecio* is named after the town of Tassa (Moquegua Region), downslope of Pirhuani peak where the species was found.

#### Discussion.

*Senecio tassaensis* appears to be closely related to *Senecio moqueguensis* which grows at the same elevational range but approaches the known range of *Senecio tassaensis* within a few hundred metres. *Senecio moqueguensis* is generally distinctive in the series for its larger size, attaining dense ground mats, and for its yellow corolla. *Senecio tassaensis* has 12–16 phyllaries (vs. 9–12), an involucre length of 6–8 mm and achene length of 1–1.2 mm, being much shorter than in *Senecio moqueguensis*. *Senecio tassaensis* is relatively a very rare species with an estimated 100 individuals known. It is less similar to *Senecio pucapampaensis*, *Senecio evacoides*, *Senecio expansus* and *Senecio repens*, and can be distinguished on the basis of the habit, trichomes, leaf shape and length, calycular bracts and phyllaries length and shape as summarized in Table 1.

#### Conservation status.

Following the criteria and categories of IUCN (2001), a preliminary status of Critically Endangered (CR) is assigned. The new species deserves protection because its total area of occupancy is less than 10 km² (ca. 5 km²) (B2); only one population is known (B2b); habitat inferred to be continuing to decline(B2b(i-iii)); population estimated to number fewer than 100 individuals (D). The suitable habitats for *Senecio tassaensis* on the mountain summits of Pirhuani peak in the Ubinas district are indicated as endangered, because changes in the annual rainfall, volcanic activity and exploitation of natural resources, may all reduce their extent.

### Key to the species of *Senecio* ser. *Suffruticosi* subser. Caespitosi in Peru

(adapted from Cabrera 1985, Cabrera et al. 1999)

**Table:** 

1a	Plants shrubby; involucres longer than 11 mm	2
1b	Plants caespitose; involucres shorter than 11 mm long	5
2a	Achenes densely pubescent; leaves 1–2 cm long, deeply dentate or lobulate	*Senecio adenophyllus*
2b	Achenes glabrous; leaves 1–3.5 cm long, entire	3
3a	Leaves 3–5 mm wide; involucre bracts oblong	*Senecio rufescens*
3b	Leaves 0.7–2 mm wide; involucre bracts linear	4
4a	Leaves 25–80 mm long; phyllaries 13–18	*Senecio scorzonerifolius*
4b	Leaves 15–25 mm long; phyllaries 15–20	*Senecio danai*
5a	Capitulum small; involucre shorter than 5 mm	6
5b	Capitulum larger; involucre shorter than 11 mm	7
6a	Leaves entire, glabrous and fleshy; phyllaries 8	*Senecio humillimus*
6b	Leaves entire or dentate, glabrous or lanuginose; phyllaries 13	*Senecio vegetus*
7a	Plants tomentose, at least on the underside of leaves	8
7b	Plants glabrous	13
8a	Plants with dense pubescence covering all plant parts	9
8b	Plants with sparse pubescence not covering all plant parts	11
9a	Leaves spathulate, 10–20 mm long; involucre 7–8 mm tall; phyllaries 13–20	*Senecio evacoides*
9b	Leaves ovate, elliptic or circular, crenate, 10–65 mm long; involucre 6–10 mm tall; phyllaries 13–25	10
10a	Involucre 10–25 mm long; phyllaries 20–25	*Senecio expansus*
10b	Involucre 6–7 mm long; phyllaries 13–20	*Senecio repens*
11a	Leaves cuneiform, lamina glabrous except puberulous margins	*Senecio pucapampaensis*
11b	Leaves oblong, lamina with trichomes on surfaces and margins	12
12a	Leaves 8–12 mm long, lamina oblong-spathulate; involucre 7–10 mm; phyllaries 9–12	*Senecio moqueguensis*
12b	Leaves 6–9 mm long, lamina obovate-spathulate; involucre 6–8 mm; phyllaries 12–16	*Senecio tassaensis*
13a	Leaves dentate, linear-cuneiform	*Senecio trifurcifolius*
13b	Leaves entire	14
14a	Leaves 10–35 mm long	*Senecio algens*
14b	Leaves less than 14 mm long	15
15a	Leaves 8–12 mm, linear-lanceolate; involucre 8–11 mm; phyllaries 6–8	*Senecio gamolepis*
15b	Leaves 9–14 mm, obovate-spathulate; involucre 7–9 mm; phyllaries 12–14	*Senecio sykorae*

## Supplementary Material

XML Treatment for Senecio moqueguensis

XML Treatment for Senecio sykorae

XML Treatment for Senecio tassaensis
